# Bilateral steroid-induced glaucoma following monocular steroid therapy: a case report

**DOI:** 10.1186/s12886-026-04829-2

**Published:** 2026-04-15

**Authors:** Xianyao Peng, Xiaoping Xu, Wuliang Li

**Affiliations:** Ningbo Key Laboratory of Medical Research on Blinding Eye Diseases, Ningbo Eye Institute, Ningbo Eye Hospital, Wenzhou Medical University, No. 599, Beimingcheng Road, Yinzhou District, Ningbo City, Zhejiang Province People’s Republic of China

**Keywords:** Steroid-induced glaucoma, Steroid eye drops, Keratoplasty

## Abstract

**Background:**

Following keratoplasty, the use of steroids is typically necessary to control postoperative inflammation and prevent graft rejection. However, improper management can lead to elevated intraocular pressure (IOP) and glaucoma. In our view, the use of topical steroid eye drops in one eye is unlikely to cause bilateral steroid-induced glaucoma.

**Case presentation:**

An 18-year-old male with a history of keratoconus in both eyes and deep anterior lamellar keratoplasty (DALK) in the right eye. Postoperatively, the patient was prescribed 0.1% fluorometholone three times daily in the right eye, and elevated IOP was detected in both eyes. After three months of treatment, glaucomatous damage advanced, and anterior segment examination revealed posterior subcapsular cataract in both eyes. IOP remained uncontrolled and a diagnosis of bilateral steroid-induced glaucoma was established.

**Conclusion:**

Monocular administration of topical steroids may be associated with bilateral steroid-induced glaucoma in susceptible individuals. We need to emphasize the importance of close monitoring and early intervention for patients taking steroid eye drops.

## Background

Secondary glaucoma is a common complication following keratoplasty, particularly when steroid drugs are used to manage postoperative inflammation and prevent graft rejection [1]. While the use of steroid drugs is often necessary, improper management can lead to elevated intraocular pressure (IOP) and glaucoma. Some studies have shown that the incidence of secondary glaucoma after surgery is in the range of 11% to 50% [2]. In our view, the use of topical steroid eye drops in one eye usually results in monocular steroid-induced glaucoma. We recently encountered a teenager with bilateral steroid-induced glaucoma following the use of topical steroids in only one eye. To our knowledge, this report presents a rare case.

## Case presentation

This case presents a patient who developed bilateral steroid-induced glaucoma after monocular use of topical steroid eye drops. An 18-year-old male, was referred to the glaucoma clinic after elevated IOP was detected during a post-keratoplasty follow-up visit for his right eye. A detailed ocular history was obtained, specifically excluding prior episodes of elevated intraocular pressure, glaucoma or glaucoma suspicion, a family history of glaucoma, ocular trauma, and uveitis. The patient denied any history of vernal keratoconjunctivitis or allergic conjunctivitis. The patient also reported no history of long-term topical or systemic steroid use, including for conditions such as allergic rhinitis or asthma that would typically require intranasal or inhaled steroids. His ophthalmic history was significant for keratoconus in both eyes, and deep anterior lamellar keratoplasty (DALK) in the right eye 9 months prior. The patient’s preoperative best-corrected visual acuity (BCVA) was 20/200 in the right eye and 20/100 in the left. IOP measured 15 mmHg in the right eye and 14 mmHg in the left eye, with corresponding cup-to-disc ratios of 0.5 and 0.6, respectively. Neither visual field testing nor optical coherence tomography (OCT) of the optic disc was performed prior to DALK due to the absence of clinical suspicion for glaucoma at that time. Topical steroid therapy (prednisolone acetate) was initiated postoperatively at a frequency of every 2 h, with a subsequent reduction to four times daily starting on the third postoperative day, without adjunctive oral steroids. At the 2-week postoperative follow-up, the topical steroid was switched from prednisolone acetate four times daily to 0.1% fluorometholone three times daily. During the 6-month post-DALK follow-up, the BCVA was 20/200 in the right eye and 20/100 in the left eye. The IOP remained within the range of 13 to 18 mmHg in the right eye and 11 to 16 mmHg in the left eye (Table 1).

**Fig. 1:**
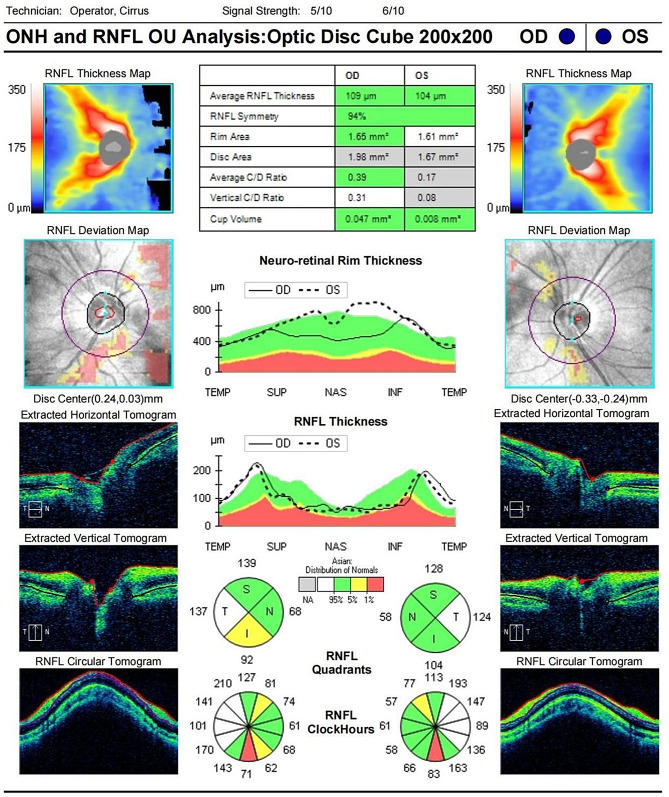
Optic coherence tomography (OCT) of the retinal nerve fiber layer (RNFL) at 9 months post-keratoplasty, showed no significant RNFL thinning in both eyes

**Fig. 2:**
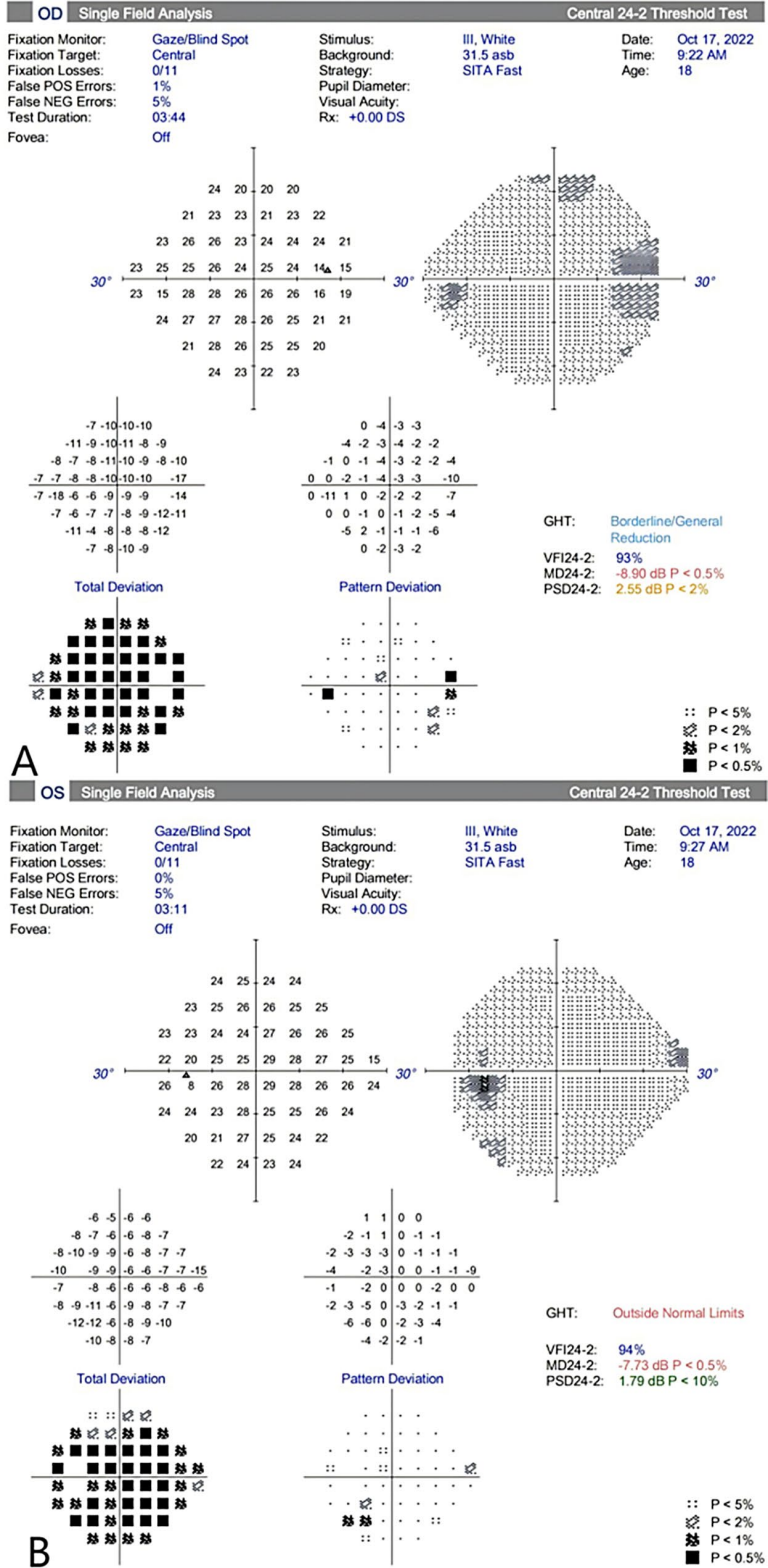
Visual field on standard automated perimetry using the SITA 24 − 2 Fast algorithm at 9 months post-keratoplasty, showed no significant defects in the right eye (**A**) and in the left eye (**B**). The mean deviations were − 8.90 dB and − 7.73 dB in the right and left eye, respectively

**Fig. 3:**
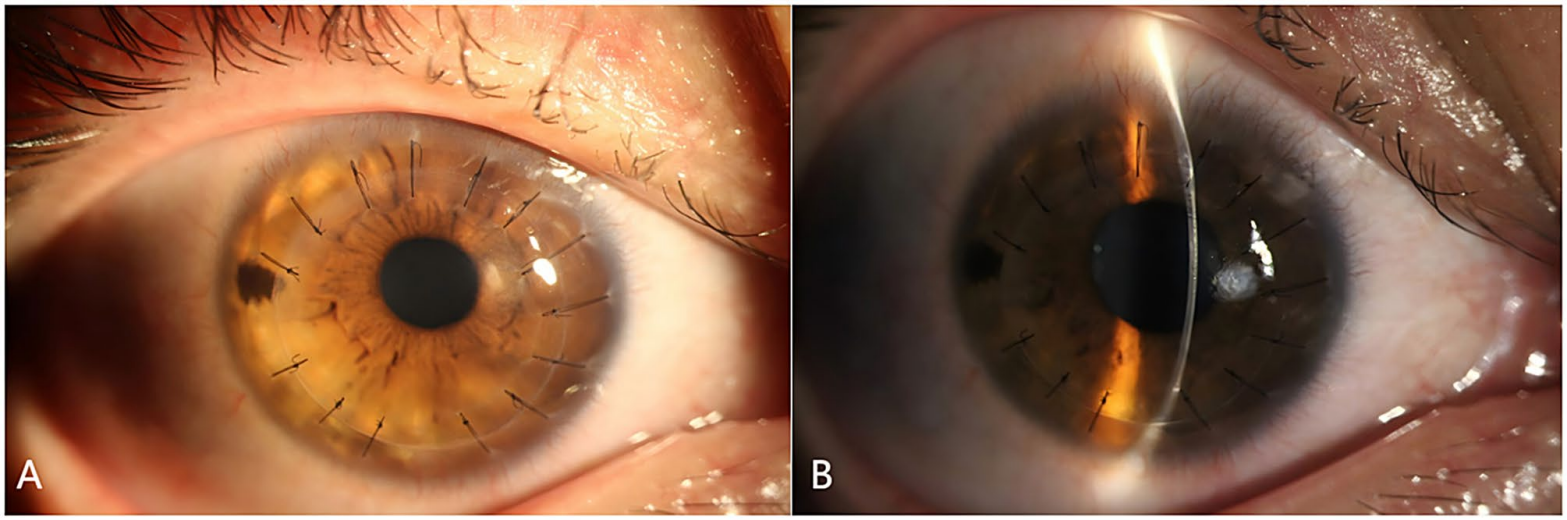
Anterior segment photographs of the right eye at 9 months post-keratoplasty, revealed a clear corneal graft (**A**) and a clear posterior lens capsule (**B**)

**Fig. 4:**
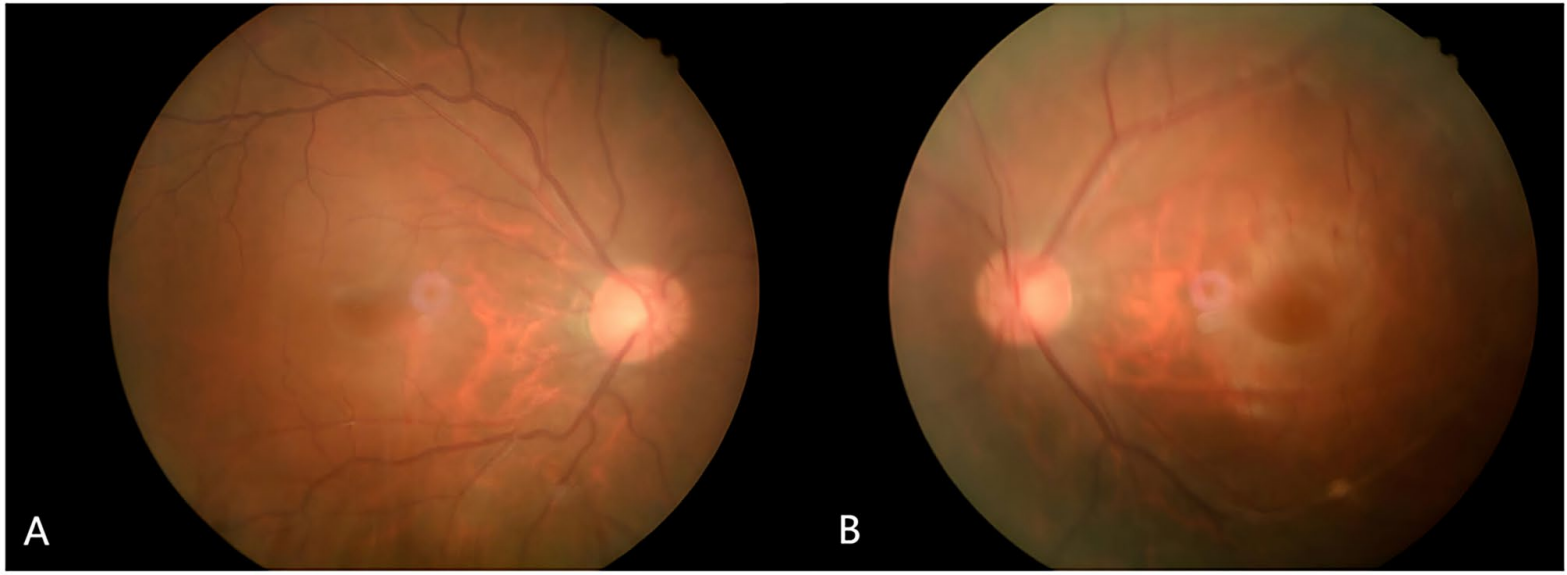
Fundus photographs at 9 months post-keratoplasty, revealed a cup-to-disc ratio of 0.5 in the right eye (**A**) and 0.6 in the left eye (**B**)

His IOP had remained within normal until the 9-month post-DALK follow-up. His retinal nerve fiber layer (RNFL) OCT showed RNFL thickness of 109 μm in the right eye and 104 μm in the left eye, with no significant RNFL thinning in both eyes (Fig. 1). His standard automated perimetry using the SITA 24 − 2 algorithm showed mean deviation values of -8.90 dB in the right eye and − 7.73 dB in the left eye (Fig. 2). On examination, his BCVA was 20/200 in the right eye and 20/100 in the left eye. His IOP was 54 mmHg in the right eye and 44 mmHg in the left eye. Gonioscopy revealed wide-open angles up to the ciliary body band without any pigmentation in both eyes. Anterior segment examination revealed a clear corneal graft in the right eye (Fig. 3). Posterior segment examination revealed a cup-to-disc ratio of 0.5 in the right eye and 0.6 in the left eye (Fig. 4). Initial management involved discontinuing the 0.1% fluorometholone and starting carteolol twice daily in both eyes, brinzolamide twice daily in both eyes, and oral acetazolamide (250 mg) twice daily.

**Fig. 5:**
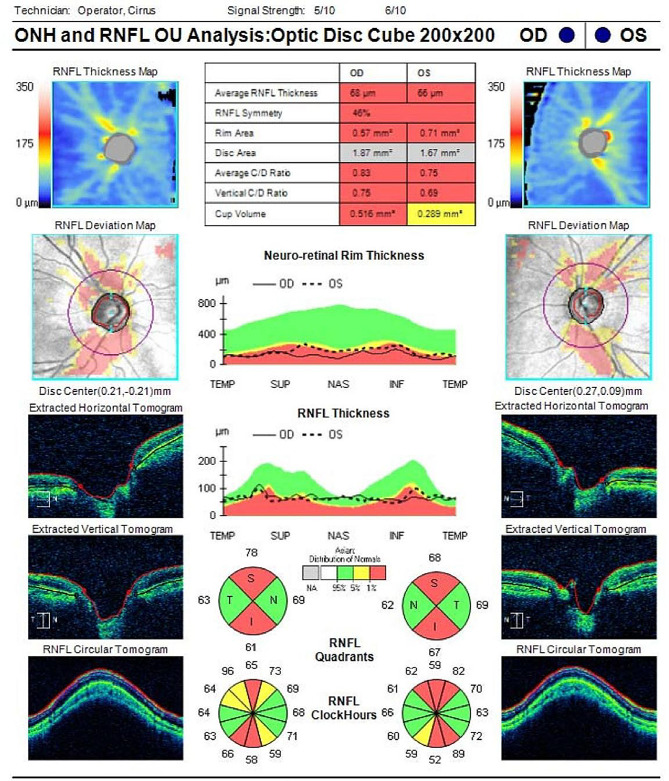
Optic coherence tomography (OCT) of the retinal nerve fiber layer (RNFL) at 12 months post-keratoplasty, demonstrating superior and inferior RNFL thinning of both eyes consistent with glaucomatous damage

**Fig. 6:**
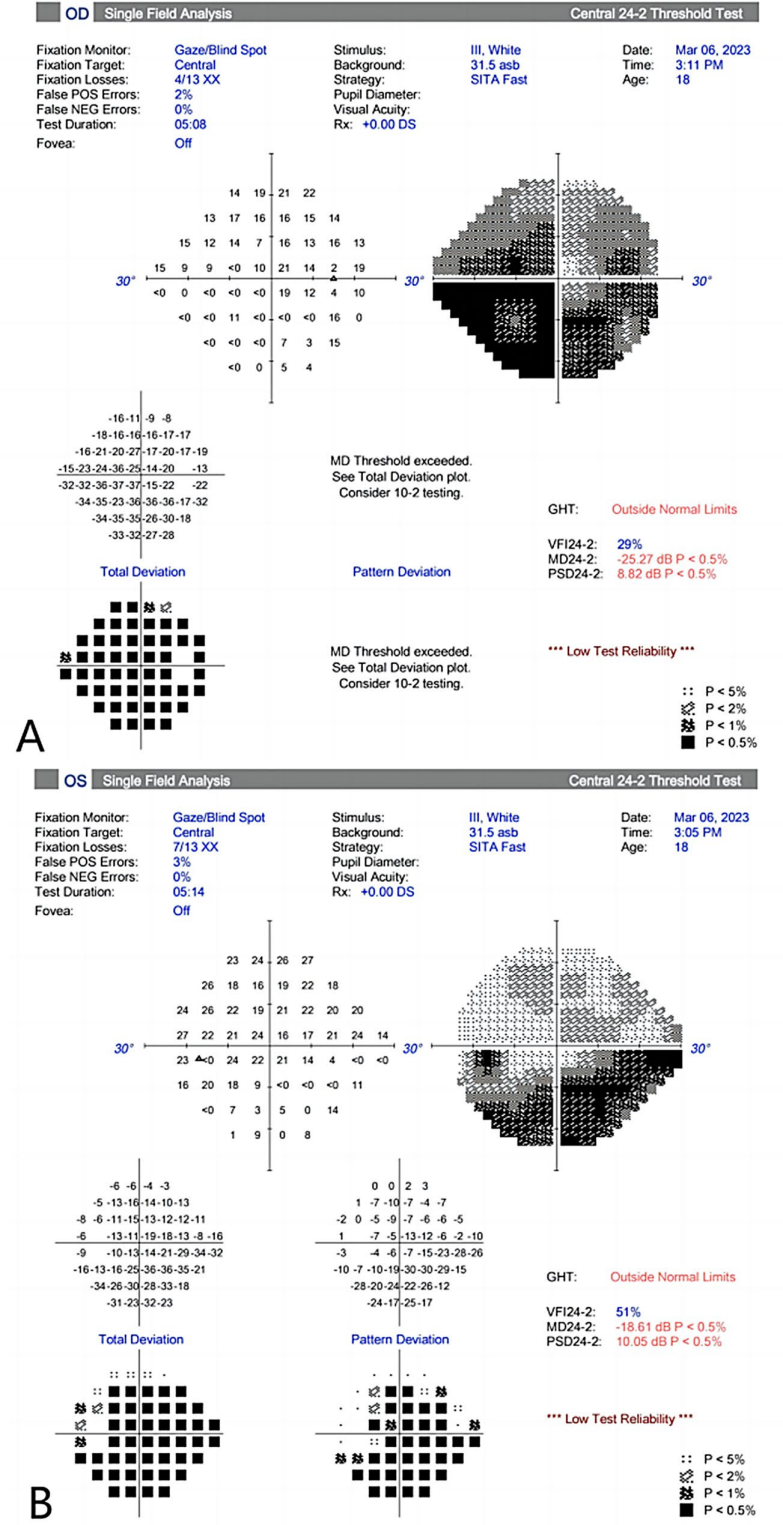
Visual field on standard automated perimetry using the SITA 24 − 2 Fast algorithm (High rate of fixation losses, resulting in low test reliability) at 12 months post-keratoplasty, demonstrated generalized depression in the right eye (**A**). And dense inferior arcuate defects in the left eye (**B**). The mean deviations were − 25.27 dB and − 18.61 dB in the right and left eye, respectively

**Fig. 7:**
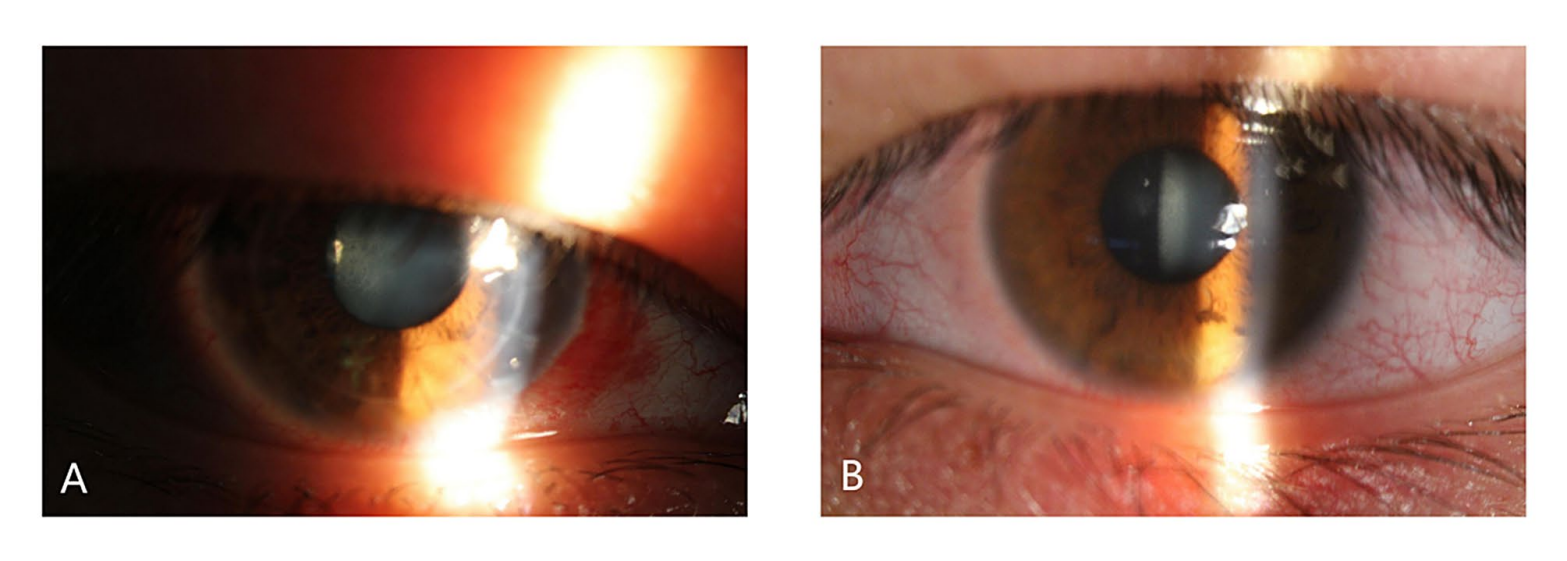
Anterior segment photographs of the right (**A**) and left (**B**) eyes at 12 months post-keratoplasty, demonstrating significant posterior subcapsular cataract in both eyes, and a clear corneal graft in the right eye

**Fig. 8:**
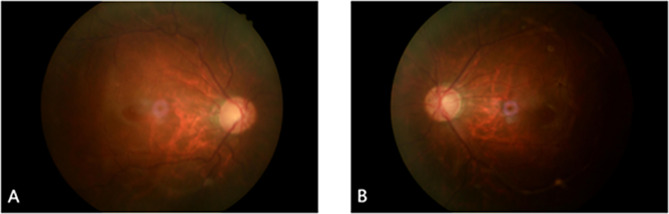
Fundus photographs of the right (**A**) and left (**B**) eyes at 12 months post-keratoplasty, demonstrating significant cupping in both eyes consistent with glaucomatous changes

After three months of treatment, the IOP remained uncontrolled and the BCVA decreased (Table 1). His OCT RNFL showed RNFL thickness of 68 μm in the right and 66 μm in the left eyes, with thinning of the superior and inferior RNFL in each eye (Fig. 5). His standard automated perimetry using the SITA 24 − 2 algorithm showed mean deviation values of -25.27 dB in the right eye and − 18.61 dB in the left eye (Fig. 6). This indicates a general visual field defect in both eyes. On examination, his BCVA was reduced to 20/400 in both eyes. His IOP was 44 mmHg in the right eye and 42 mmHg in the left eye. Gonioscopy revealed wide-open angles up to the ciliary body band without any pigmentation in both eyes. Anterior segment examination revealed a clear corneal graft in the right eye and posterior subcapsular cataract in both eyes (Fig. 7). Posterior segment examination revealed a cup-to-disc ratio of 0.8 in both eyes (Fig. 8). Based on the clinical history and findings, a diagnosis of bilateral steroid-induced glaucoma was established.

Given the high IOP and the severe glaucomatous damage, canaloplasty was performed in both eyes. On the first postoperative day, his IOP was 12 mmHg in the right eye and 11 mmHg in the left eye. During the 6-month postoperative period, his average IOP was 14 mmHg (11–17 mmHg) in the right eye and 15 mmHg (13–18 mmHg) in the left eye without any anti-glaucoma medicine. His BCVA was 20/400 in the right eye and 20/400 in the left eye. The patient reported relief after surgery due to stable intraocular pressure and expressed satisfaction with the surgical outcome, despite limited visual recovery.

**Table 1:** Prescription, best-corrected visual acuity and intraocular pressure change over time

Follow up	Prescription	OD		OS	
		BCVA	IOP fluctuation range (mmHg)	BCVA	IOP fluctuation range (mmHg)
Pre-DALK	-	20/200	15	20/100	14
6-month post-DALK	0.1% fluorometholone	20/200	13–18	20/100	11–16
9-month post-DALK	-	20/200	54	20/100	44
1-month after treatment	Carteolol, brinzolamide, acetazolamide tablets,	20/200	27–59	20/100	21–44
2-month after treatment	Carteolol, brinzolamide, tafluprost, pilocarpine, acetazolamide tablets,	20/200	37–57	20/200	37–38
3-month after treatment	Brinzolamide and brimonidine, latanoprost, acetazolamide tablets	20/400	37–51	20/400	28–42
6-month post-canaloplasty	-	20/400	11–17	20/400	13–18

OD: Right eye, OS: left eye, BCVA: Best-corrected visual acuity, IOP: Intraocular pressure, DALK: Deep anterior lamellar keratoplasty

### Discussion and conclusion

The patient’s glaucoma and cataract development are attributed to prolonged use of steroid eye drops following corneal transplantation, which led to severe, irreversible visual impairment. Steroid-induced glaucoma is a recognized complication post-keratoplasty, and is a form of open angle glaucoma. Steroid-induced glaucoma is primarily attributed to impaired aqueous humor outflow through the trabecular meshwork. This impairment is presumably caused by the deposition of elevated levels of extracellular matrix molecules, including collagen, fibronectin, among others [3,4].

Interestingly, this case is unique in its bilateral presentation of steroid-induced glaucoma following monocular steroid administration. 

This phenomenon may be similar to the contralateral reduction in IOP observed with topical β-blockers such as timolol, which is believed to be mediated by systemic absorption of the drug, first through the nasolacrimal mucosa and then via the bloodstream to the contralateral eye [5,6]. It is speculated that the patient may have been in a preclinical stage of primary open-angle glaucoma (POAG) without initial IOP elevation, where the trabecular meshwork had already undergone pathological changes [7]. The administration of steroid eye drops likely exacerbated these changes, advancing the patient from a preclinical to a clinical POAG stage with ocular hypertension [8].

Several studies have found that steroid-induced glaucoma typically develops 3 to 6 months after keratoplasty, with a longer duration of steroid use being associated with a higher risk of intraocular hypertension [9]. Both DALK and penetrating keratoplasty (PK) necessitate postoperative steroid therapy and are therefore associated with a risk of steroid-induced ocular hypertension and glaucoma. While PK has historically been associated with a higher incidence of secondary glaucoma due to factors such as full-thickness surgery, angle distortion, and prolonged steroid use, current evidence does not demonstrate a higher risk of steroid-induced glaucoma following DALK compared to PK. Instead, the development of elevated intraocular pressure appears to be more closely related to the potency, duration, and frequency of steroid therapy, as well as individual susceptibility, rather than the keratoplasty technique itself [10]. For patients requiring long-term steroid therapy, especially those using topical medications and sensitive to steroid drugs, the choice of medication is important. Topical steroid drugs are more likely to induce glaucoma [11,12]. Therefore, it is crucial to choose drugs that have a lower potential to elevate IOP. The risk of increased IOP due to steroid drugs is closely linked to corneal permeability, drug concentration, and duration of use. Medications with higher corneal permeability present a greater risk of inducing elevated IOP. Thus, for long-term treatment, selecting low-concentration drugs with poor corneal permeability is advisable. 

It is essential to routinely monitor IOP and assess for any changes. Patients with steroid-induced intraocular hypertension or glaucoma should be closely followed to evaluate their response to treatment and prevent further optic nerve damage. Goñi et al. [13] suggested the following IOP monitoring protocol: (1) Measure baseline IOP and assess for glaucomatous damage. (2) Evaluate risk factors, including IOP ≥ 15 mmHg, family history of glaucoma, prior steroid-induced IOP elevation, and current steroid use. (3) If IOP > 21 mmHg, perform visual field testing, imaging of the optic nerve and RNFL. Delay treatment and follow up within 6 weeks. (4) If IOP > 25 mmHg, initiate treatment with medications or laser intervention, perform visual field testing and imaging of the optic nerve and RNFL, and conduct follow-up within 6 weeks. If IOP remains > 25 mmHg and has received ≥ 2 medications, refer the patient to a glaucoma specialist.

The management of steroid-induced glaucoma parallels that of POAG, including topical anti-glaucoma medications, laser trabeculoplasty, filtration surgery, and glaucoma drainage implant surgery [14]. When medical management fails to control the IOP, selective laser trabeculoplasty (SLT) can be tried in, SLT has shown efficacy in reducing IOP and dependency on medications in such cases [15, 16]. Recent researches also support the effectiveness and safety of goniotomy [17]. As demonstrated in this patient, canaloplasty effectively reduced IOP without serious complications, consistent with the findings of Paolo Brusini’s study [18]. The favorable IOP reduction observed after canaloplasty in this case prompts consideration of earlier surgical intervention for steroid-induced glaucoma refractory to medical therapy. In selected patients, particularly those with persistently elevated IOP on maximally tolerated medication, rapid glaucomatous progression, or poor adherence, earlier surgery may be justified. However, given the potentially reversible nature of steroid-induced ocular hypertension upon discontinuation of steroids, medical therapy remains first-line [19]. Therefore, surgical intervention should be reserved for cases demonstrating inadequate IOP control or progressive damage despite optimized medical management.

This case underscores the potential for severe bilateral glaucoma induced by monocular steroid use. The patient’s rapid progression from steroid-induced ocular hypertension to steroid-induced glaucoma highlights the importance of close monitoring and early intervention.

## Data Availability

Data is available upon request made to the corresponding author.

## Abbreviations

IOP: Intraocular pressure
DALK: Deep anterior lamellar keratoplasty
OCT: Optical coherence tomography
RNFL: Retinal nerve fiber layer
BCVA: Best-corrected visual acuity
POAG: Primary open-angle glaucoma
PK: Penetrating keratoplasty
SLT: Selective laser trabeculoplasty
